# Activity-Based Prospective Memory and Motor Sleep Inertia in Insomnia

**DOI:** 10.3390/brainsci14121248

**Published:** 2024-12-12

**Authors:** Lorenzo Tonetti, Miranda Occhionero, Sara Giovagnoli, Federica Giudetti, Elena Briganti, Vincenzo Natale

**Affiliations:** 1 Department of Psychology “Renzo Canestrari”, University of Bologna, 40127 Bologna, Italy; miranda.occhionero@unibo.it (M.O.); sara.giovagnoli@unibo.it (S.G.); federica.giudetti3@unibo.it (F.G.); vincenzo.natale@unibo.it (V.N.); 2 Pediatric Neuromuscular Unit, IRCCS Institute of Neurological Sciences, 40100 Bologna, Italy; elena.briganti91@gmail.com

**Keywords:** prospective memory, insomnia, actigraphy, sleep inertia

## Abstract

Background/Objectives: The aim of this study is to shed light on activity-based prospective memory upon the awakening and its association with motor sleep inertia in different phenotypes of insomnia disorder. Methods: To this end, 67 patients with insomnia and 51 healthy controls took part in the study. After enrollment, previously proposed actigraphic quantitative criteria were adopted, and the following phenotypes of insomnia disorder were observed in the patient sample: sleep onset (*n* = 12), maintenance (*n* = 19), mixed (*n* = 17), and negative misperception (*n* = 19). Each participant had used the Micro Motionlogger Watch (Ambulatory Monitoring, Inc., Ardsley, NY, USA) actigraph for one week. Actigraphic recording allowed for a description of both the activity-based prospective memory performance upon the awakening—by computing the time interval between sleep end and the time participants actually remembered to push the event-marker button of the actigraph—and the motor sleep inertia, i.e., the mean motor activity, minute-by-minute, in the first 60 min after sleep end in the morning. Results: Compared to healthy controls, a longer time interval was observed between sleep end and activity-based prospective memory performance in patients with mixed and maintenance insomnia. Moreover, a significant association was highlighted between motor sleep inertia and the activity-based prospective memory performance: higher levels of motor activity in those who remembered to perform the memory task early after sleep end, that spread over a longer time interval in maintenance and mixed insomnia. Conclusions: Overall, the present results seem to highlight a more marked cognitive inertia in patients with mixed and maintenance insomnia as well as a significant association between motor and cognitive inertia that spreads over a different time interval according to the phenotype of insomnia.

## 1. Introduction

Prospective memory can be conceived as the ability to remember to perform a planned action or recall a planned intention at some future point in time. This type of memory includes remembering what and when an activity must be performed (retrospective component) and requires us to remember to execute the intended action at the appropriate moment (prospective component). Based on the execution modality, Kvavilashvili and colleagues [1] differentiated between three types of prospective memory: time-based (the intention to perform an action that should be executed at a specific time or after a specific amount of time), event-based (the intention to perform an action, which is activated by an external cue), and activity-based (the intention is executed prior to or after a specific activity). Regarding the methods of prospective memory assessment, the ecological validity of the tasks used in a laboratory setting was questioned [2,3] because these do not cover the main prospective memory requests encountered in daily life [4].

In order to investigate the functioning of activity-based prospective memory in an ecological manner, some authors [5] introduced a new research paradigm: subjects were asked to remember to push the event-marker button of an actigraph (a device that is able to directly record motor activity and indirectly assess sleep, and that is usually worn around the non-dominant wrist) when they went to bed and were trying to fall asleep and when they woke up in the morning. This ecological assessment of activity-based prospective memory (i.e., an assessment able to cover the main requests of prospective memory in daily life) was later used by other researchers [6,7] and it proved to be a useful methodology for investigating the process of transitioning from sleep to wakefulness: sleep inertia. This term refers to the transitional phase characterized by reduced arousal immediately after waking from sleep, leading to a slow reactivation of cognitive and motor performance. This impairment is due to the gradual reactivation of cognitive functions and can vary in duration, typically lasting from a few minutes to several hours depending on various factors such as sleep stage at awakening and individual differences [8].

The field of the naturalistic investigation of activity-based prospective memory performance in association with motor sleep inertia (i.e., the motor activity, minute-by-minute, in the first 60 min after sleep end) has recently been studied. Occhionero et al. [9] recently carried out a retrospective study in children and adult patients with attention deficit hyperactivity disorder (ADHD), compared with healthy children and adults, computing the efficiency of activity-based prospective memory as the time interval in minutes between sleep end and the time at which participants remembered to push the event-marker button of the actigraph. Regardless of age, ADHD patients took more time to perform the prospective memory task than healthy controls. Authors also examined the potential association between activity-based prospective memory performance and motor sleep inertia; a significant association was observed in only the healthy children, with those who remembered to push the event-marker button closer to sleep end being more active in the first 8 min after awakening.

Even more recently, in another disorder, insomnia, Occhionero et al. [10] examined the efficiency of activity-based prospective memory performance in a slightly different way, that is, by computing the ratio between the number of times the participant remembered to push the event-marker button of the actigraph after awakening and the number of recording nights multiplied by 100. Some well-established diagnostic criteria for insomnia disorder are available, as those reported in the third edition of the International Classification of Sleep Disorders (ICSD-3) [11]. In that study, Occhionero et al. [10] reached a diagnosis of chronic insomnia disorder, during a clinical interview, if the criteria according to the ICSD-3 were fulfilled as, for example, the sleep disturbance and associated daytime symptoms occurred for at least three times per week and for at least three months. However, in order to reach a phenotypical classification of insomnia not potentially biased by the patients’ reports, Occhionero et al. [10] chose to adopt an objective criterion based on the output of an actigraphic recording. More specifically, according to the quantitative actigraphic criteria put forward by Natale and colleagues [12] for the assessment of insomnia, Occhionero et al. [10] took into account the following four different phenotypes of insomnia: sleep onset (only the parameter of sleep onset latency was higher than the corresponding cut-off value), maintenance (wake after sleep onset and/or the number of long wake episodes above the corresponding cut-off values), mixed (both sleep onset latency and wake after sleep onset or long wake episodes outside normal limits), and negative misperception (the complete set of actigraphic parameters within normal limits) insomnia. The authors [10] showed that patients with maintenance and mixed insomnia have impaired activity-based prospective memory performance compared with healthy controls, while patients with sleep onset and negative misperception insomnia showed similar memory performance to controls. However, this study did not examine the cognitive sleep inertia (in our context, the term refers to the functional restructuring of the cognitive system, which must restore the waking cognitive processes from the sleep modality), i.e., the time interval between sleep end and the time at which participants performed the prospective memory task—nor its association with motor sleep inertia, as previously carried out with reference to ADHD.

To fill this observational gap, the aim of this work is to shed light on activity-based prospective memory upon the awakening and its association with motor sleep inertia in different phenotypes of insomnia disorder.

## 2. Materials and Methods

### 2.1. Participants

Sixty-seven patients with insomnia (40 females; mean age ± SD = 38.13 ± 15.68) and 51 healthy controls (25 females; mean age ± SD = 22.73 ± 3.12) were enrolled.

Following the international guidelines [11], patients were recruited from the “Servizio di Diagnosi e Cura delle Insonnie” (Service for the Diagnosis and Treatment of Insomnias) at the Department of Psychology “Renzo Canestrari” of the University of Bologna (Bologna, Italy). Patients were included in the present study only if, at the time of actigraphic recording, they did not meet the following exclusion criteria: (1) the use of hypnotic drugs and/or medications able to interfere with sleep quality; (2) the presence of physical and/or mental conditions causing the disorder. According to the quantitative actigraphic criteria put forward by Natale and colleagues [12] for the assessment of insomnia, patients were grouped according to the following phenotypes of insomnia disorder using the same procedure previously used by Occhionero et al. [10]: sleep onset insomnia, SO-I (only sleep onset latency higher than the cut-off value of 12 min) (*n* = 12, 7 females, mean age ± SD = 35.5 ± 14.68); maintenance insomnia, MA-I (wake after sleep onset and/or number of awakenings higher than 5 min above the cut-off values of 25 min and 1.8, respectively) (*n* = 19, 10 females, mean age ± SD = 37.26 ± 15.81); mixed insomnia, MIX-I (both sleep onset latency and wake after sleep onset or the number of awakenings longer than 5 min higher than the corresponding cut-off values) (*n* = 17, 10 females, mean age ± SD = 42.18 ± 17.31); and negative misperception insomnia, NM-I (the whole set of actigraphic parameters within normal limits) (*n* = 19, 13 females, mean age ± SD = 37.05 ± 15.22).

The recruitment of healthy controls (HC) took place at the Laboratory of Applied Chronopsychology at the Department of Psychology “Renzo Canestrari” of the University of Bologna (Bologna, Italy). During an interview, the absence of sleep disorders was ascertained, as well as drug and medication intake that may interfere with the physiological sleep–wake cycle.

### 2.2. Actigraphy

The actigraph model Micro Motionlogger Watch (Ambulatory Monitoring Inc., Ardsley, NY, USA) was used in the present study to measure motor activity directly, and sleep indirectly, using algorithms validated through polysomnography [13,14]. The validity of actigraphy in sleep assessment has been quite extensively documented [15,16,17].

The software Watchware (version 1.99.34.1; Ambulatory Monitoring Inc., Ardsley, NY, USA) was used to initialize the actigraphs in zero crossing mode to collect data in 1 min epochs, while the software Action W2 (version 2.7.3285; Ambulatory Monitoring Inc., Ardsley, NY, USA) and Action 4 (version 1.16; Ambulatory Monitoring Inc., Ardsley, NY, USA) were used to score sleep and extract raw motor activity, respectively.

### 2.3. Actigraphic Sleep Measures

Regarding sleep, the following measures were computed: total sleep time (TST), the sum in minutes of all sleep epochs between sleep onset and get-up time; sleep efficiency (SE), the ratio between total sleep time and time in bed multiplied by 100; sleep onset latency (SOL), the time in minutes between bedtime and sleep onset (i.e., the first epoch of a block of 20 consecutive sleep epochs with no more than 1 wakefulness epoch); wake after sleep onset (WASO), the sum in minutes of all the wakefulness epochs between sleep onset and get-up time; number of awakenings lasting more than 5 min (NA > 5); and mean motor activity (MA, mean number of movements in 1 min epochs) during the time spent in bed.

### 2.4. Inertia of Activity-Based Prospective Memory Performance and Motor Sleep Inertia

**Figure 1:**
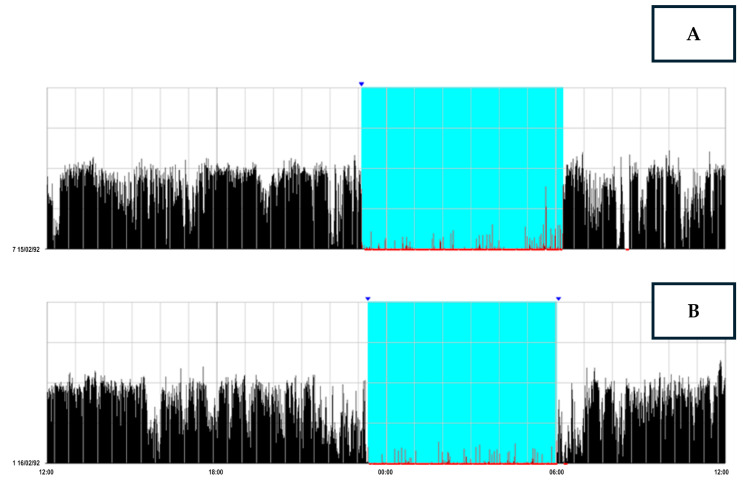
Example of an actigraphic record spanning two days, with one line representing each day. The x-axis indicates the time of day, while the y-axis represents motor activity. The dates 15/02/92 (first line) and 16/09/92 (second line) refer to the recordings’ day/month/year. The actigraphic software assigns a number to each day of the week: 1 for Sunday, 2 for Monday, 3 for Tuesday, 4 for Wednesday, 5 for Thursday, 6 for Friday, and 7 for Saturday. Therefore, the number 7 next to the date 15/09/92 indicates that it was a Saturday, while the number 1 next to 16/02/92 shows that it was a Sunday. In this figure, the black histograms represent motor activity counts recorded minute-by-minute, while the colored area indicates the time spent in bed. The red indicates that the algorithm scored the corresponding epoch as sleep. The triangle marks the moment the participant pressed the event-marker button on the actigraph. As observed in the figure, the ecological activity-based prospective memory task was performed upon waking after the second night (labeled (**B**)). In contrast, it was not performed after the awakening from the first night (labeled (**A**)).

Inertia of activity-based prospective memory performance was investigated by computing the time interval in minutes between sleep end and the time participants remembered to push the event-marker button of the actigraph, only considering the awakenings with the activity-based prospective memory task being carried out within 60 min from sleep end (Figure 1B). If the event-marker button of the actigraph was not pushed within that time interval (Figure 1A), then the corresponding awakening was discarded from statistical analyses.

Motor sleep inertia was examined by taking into account the motor activity, minute-by-minute, in the first 60 min after sleep end. If the participant removed the actigraph for any reason (e.g., to take a shower) during that time interval, then the awakening was not taken into account in the statistical analyses.

### 2.5. Procedure

Participants were requested to wear the actigraph on their non-dominant wrist for one week [18,19] and to push the event-marker button at bedtime and get-up time.

Each participant provided written informed consent before being enrolled in the study, which was approved by the Bioethics Committee of the University of Bologna (Bologna, Italy; ethical committee protocol 284,786 of 5 November 2021).

### 2.6. Statistical Analyses

In order to assess gender and age differences between groups, a chi-squared test and an ANOVA—with group as the independent variable at 5 levels (the 4 phenotypes of insomnia and healthy controls) and age as a dependent variable—were carried out, respectively.

To assess differences in sleep parameters between groups, a MANCOVA was performed with group as the independent variable at 5 levels (4 phenotypes of insomnia and healthy controls), actigraphic sleep measures as dependent variables, and age as covariate. To explore the significant effects, Tukey’s Honest Significant Difference (HSD) test for the unequal number of samples was carried out.

In order to compare the so-called cognitive sleep inertia between groups, i.e., the interval in minutes between sleep end and the time the participants remembered to perform the activity-based prospective memory task, i.e., pushing the event-marker button of the actigraph, an independent samples Kruskal–Wallis test was performed. To explore significant effects, pairwise comparisons of the group were carried out.

To explore the difference in the time course of motor sleep inertia in different phenotypes of insomnia, the statistical framework of Functional Linear Modeling (FLM) [20] was used, comparing the functional forms of motor activity, minute-by-minute, in the first 60 min after sleep end in different groups. To examine the association between the inertia of activity-based prospective memory performance and motor sleep inertia, a set of FLM analyses was separately carried out for each group by analyzing the covariation of the functional form of motor activity, minute-by-minute, in the first 60 min after sleep end against the time interval, in minutes, between sleep end and the time participants remembered to push the event-marker button of the actigraph.

## 3. Results

### 3.1. Demographics

The gender distribution across groups was not significantly different (χ^2^_4_ = 2.32; *p* = 0.68), while the age difference reached significance level (F_4,113_ = 12.59; *p* < 0.001). From Tukey’s HSD test, the mean age of HC (22.73 ± 3.12) was significantly different compared to that of the patients with NM-I (37.05 ± 15.22; *p* < 0.005), MIX-I (42.18 ± 17.31; *p* < 0.001), and MA-I (37.26 ± 15.81; *p* < 0.005).

### 3.2. Actigraphic Sleep Parameters

As shown in Table 1, significant differences were observed between groups with reference to each actigraphic sleep parameter.

To explore such differences, a set of Tukey’s HSD tests was performed showing the following significant comparisons:

- TST: MA-I ≠ NM-I (*p* < 0.05)
- SE: NM-I ≠ MIX-I (*p* < 0.001); NM-I ≠ MA-I (*p* < 0.001); MIX-I ≠ SO-I (*p* < 0.01); MIX-I ≠ HC (*p* < 0.001); MA-I ≠ SO-I (*p* < 0.05); MA-I ≠ HC (*p* < 0.001)
- SOL: NM-I ≠ MIX-I (*p* < 0.001); NM-I ≠ SO-I (*p* < 0.001); MIX-I ≠ MA-I (*p* < 0.001); MI-I ≠ HC (*p* < 0.001); MA-I ≠ SO-I (*p* < 0.005).
- WASO: NM-I ≠ MIX-I (*p* < 0.001); NM-I ≠ MA-I (*p* < 0.001); MIX-I ≠ SO-I (*p* < 0.005); MIX-I ≠ HC (*p* < 0.001); MA-I ≠ SO-I (*p* < 0.005); MA-I ≠ HC (*p* < 0.001)
- NA > 5: NM-I ≠ MIX-I (*p* < 0.001); NM-I ≠ MA-I (*p* < 0.001); MIX-I ≠ SO- I (*p* < 0.005); MIX-I ≠ HC (*p* < 0.001); MA-I ≠ SO-I (*p* < 0.001); MA-I ≠ HC (*p* < 0.001)
- MA: NM-I ≠ MIX-I (*p* < 0.005); NM-I ≠ MA-I (*p* < 0.001); MIX-I ≠ HC (*p* < 0.001); MA-I ≠ HC (*p* < 0.001)

### 3.3. Inertia of Activity-Based Prospective Memory Performance

**Figure 2:**
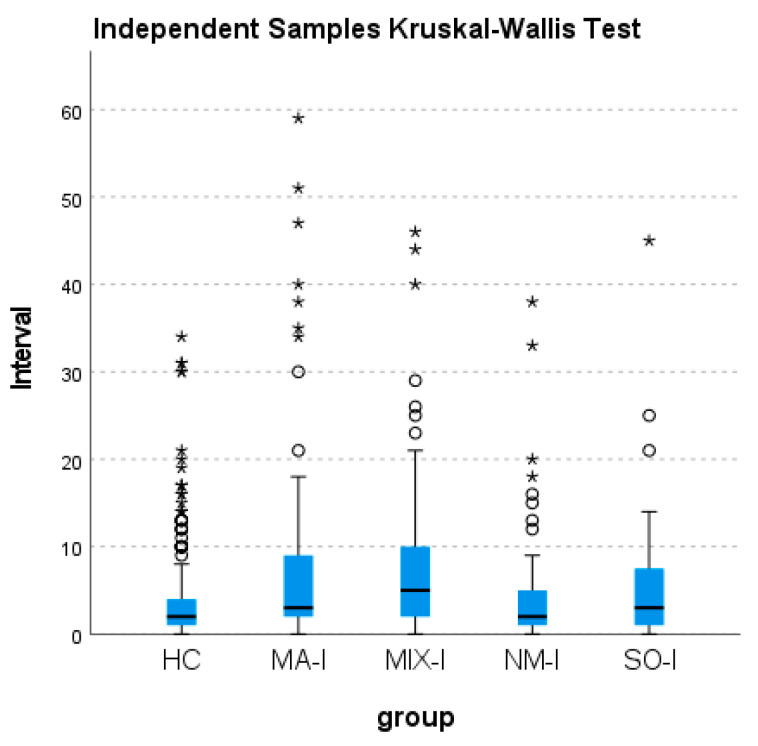
Boxplots of the time interval (in minutes) between sleep end and the time at which participants remembered to perform the activity-based prospective memory task, in the healthy controls (HC), the patients with maintenance (MA-I), mixed (MIX-I), negative misperception (NM-I), and SO-I (sleep onset) insomnia. The boxes represent the interquartile ranges (IQR), that is, the range between the first quartile (Q1) and third quartile (Q3); the central line is the median (Q2). Whiskers indicate the dispersion of values below the first and above the third quartile that were not classified as outliers (1.5 × IQR from the edge of the box). The circles represent the mild outliers (Q3 + 1.5 × IQR), and the stars represent the extreme outliers (Q3 + 3 × IQR).

As reported in Figure 2, the inertia of activity-based prospective memory performance was significantly different between the groups (χ^2^_4_ = 28.61; *p* < 0.001). When the pairwise comparisons were performed, it emerged that the time taken by HC to perform the prospective memory task after sleep end was significantly lower than that taken by patients with MIX-I (test statistics = −75.54; standard error = 18.82; standard test statistic = −4.01; adjusted significance value = 0.001) and MA-I (test statistics = −56.40; standard error = 17.81; standard test statistic = −3.17; adjusted significance value = 0.015).

### 3.4. Motor Sleep Inertia

**Figure 3:**
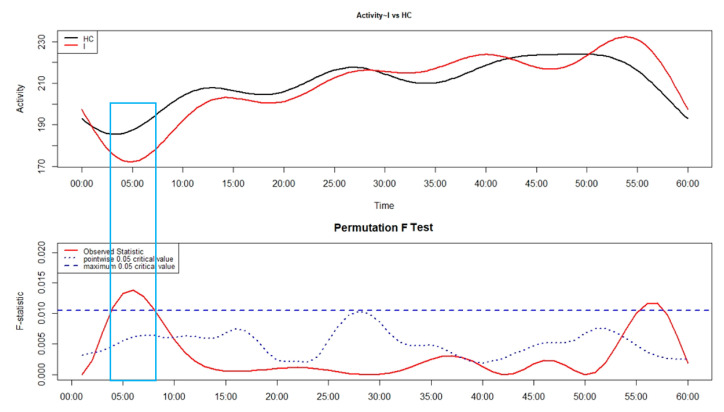
An FLM plot showing—in the upper panel—the functional forms of motor activity, minute-by-minute, over the first 60 min after sleep end in the patients with insomnia (I, red line) and in the healthy controls (HC, black line). The lower panel shows the results of the non-parametric permutation F test, with significant results when the observed statistic (red solid line) is above the global (blue dashed line) and point-wise (blue dotted line) tests of significance. The rectangle highlights the time window with significant differences between I and HC.

As shown in Figure 3, the group of patients, regardless of the phenotype of insomnia (I), showed significantly lower levels of motor activity than HC between around 4 and 8 min after sleep end.

**Figure 4:**
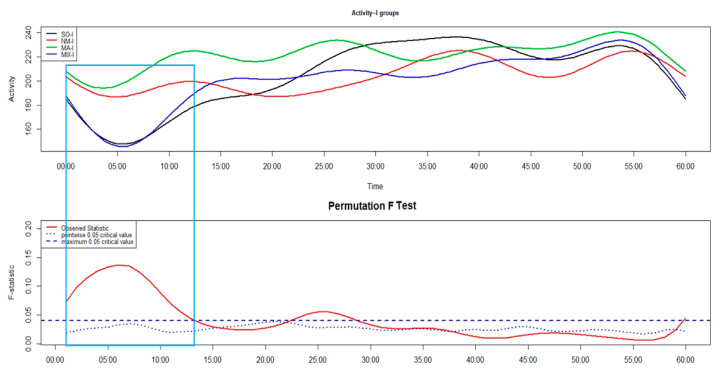
In the upper panel of the FLM plot, the functional forms of motor activity are depicted, minute-by-minute, over the first 60 min after sleep end in patients with sleep onset insomnia (SO-I, black line), negative misperception insomnia (NM-I, red line), maintenance insomnia (MA-I, green line), and mixed insomnia (MIX-I, blue line). The lower panel shows the results of the non-parametric permutation F test. The rectangle highlights the time window with significant differences among different insomnia phenotypes.

Comparing the motor sleep inertia between different phenotypes of insomnia (Figure 4), MA-I and NM-I had higher levels of motor activity than MIX-I and SO-I in around the first 13 min after sleep end.

### 3.5. Motor Sleep Inertia and Inertia of Activity-Based Prospective Memory Performance

**Figure 5:**
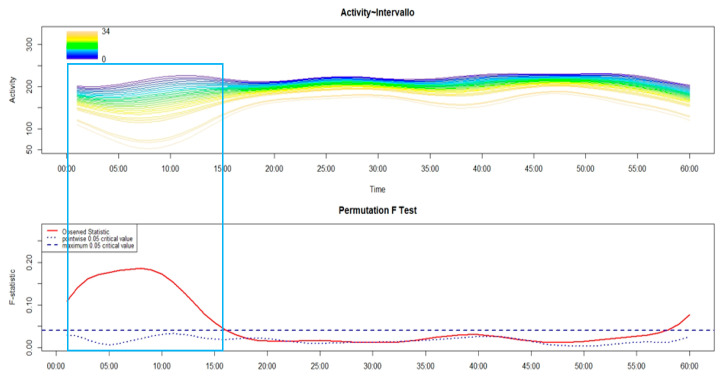
The upper panel of the FLM plot shows the functional forms of motor activity, minute-by-minute, over the first 60 min after sleep end, plotted against the time interval, in minutes, between sleep end and the time at which HC remembered to perform the activity-based prospective memory task, reported in the plot’s color spectrum legend. In addition, each line of the upper panel corresponds to the motor activity profile of a single participant marked with a color that indicates the time interval in minutes taken to perform the prospective memory task after morning awakening, with the dark colors pointing to a lower time interval while the light colors show a longer time interval. The lower panel shows the results of the non-parametric permutation F test. The rectangle highlights the time window with significant differences in motor activity according to the time interval between sleep end and the activity-based prospective memory performance.

**Figure 6:**
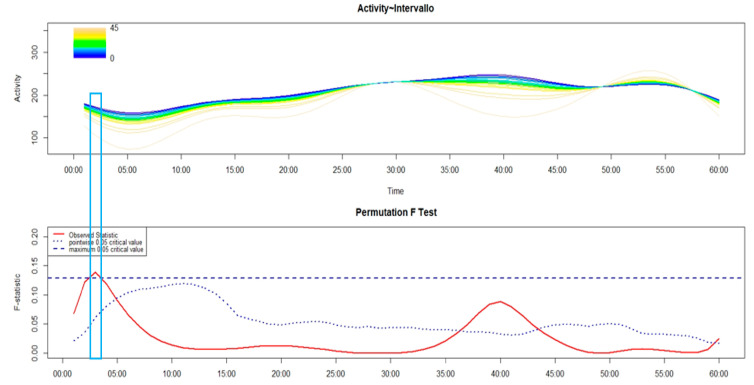
FLM plot showing—in the upper panel—the functional forms of motor activity, minute-by-minute, over the first 60 min after sleep end, plotted against the time interval, in minutes, between sleep end and the time at which patients with SO-I remembered to perform the activity-based prospective memory task, with the dark colors pointing to a lower time interval while the light colors show a longer time interval as reported in the plot’s color spectrum legend. The lower panel shows the results of the non-parametric permutation F test. The rectangle highlights the time window with significant differences in motor activity according to the time interval between sleep end and the activity-based prospective memory performance.

**Figure 7:**
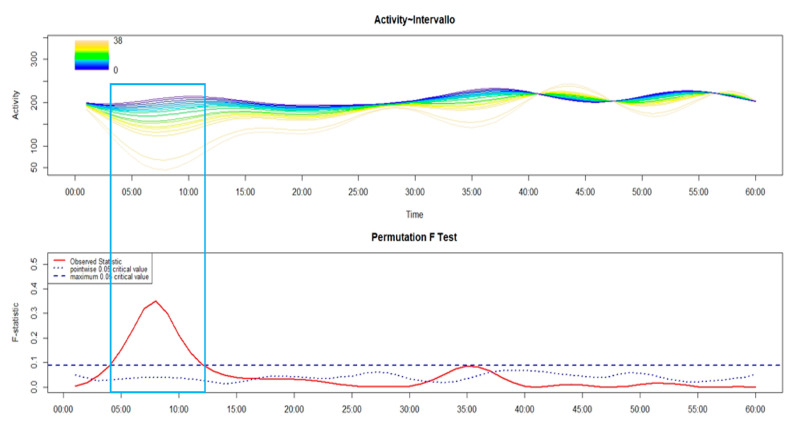
FLM plot showing—in the upper panel—the functional forms of motor activity, minute-by-minute, over the first 60 min after sleep end plotted against the time interval, in minutes, between sleep end and the time at which patients with NM-I remembered to perform the activity-based prospective memory task, with the dark colors pointing to a lower time interval while the light colors show a longer time interval as reported in the plot’s color spectrum legend. The lower panel shows the results of the non-parametric permutation F test. The rectangle highlights the time window with significant differences in motor activity according to the time interval between sleep end and the activity-based prospective memory performance.

**Figure 8:**
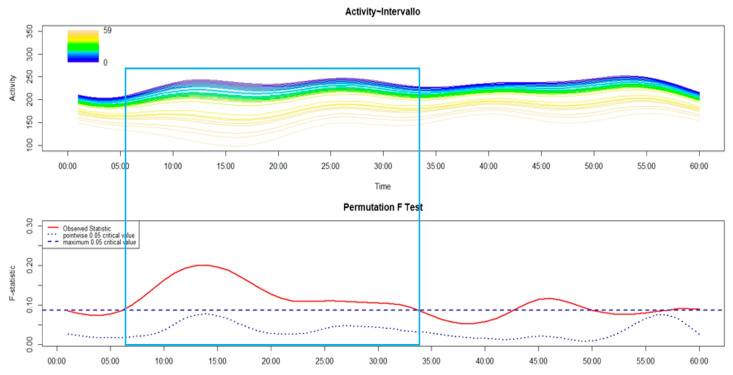
The upper panel of the FLM plot shows the functional forms of motor activity, minute-by-minute, over the first 60 min after sleep end plotted against the time interval, in minutes, between the sleep end and the time at which patients with MA-I remembered to perform the activity-based prospective memory task, with the dark colors pointing to a lower time interval while light colors show a longer time interval as reported in the plot’s color spectrum legend. The lower panel shows the results of the non-parametric permutation F test. The rectangle highlights the time window with significant differences in motor activity according to the time interval between sleep end and the activity-based prospective memory performance.

**Figure 9:**
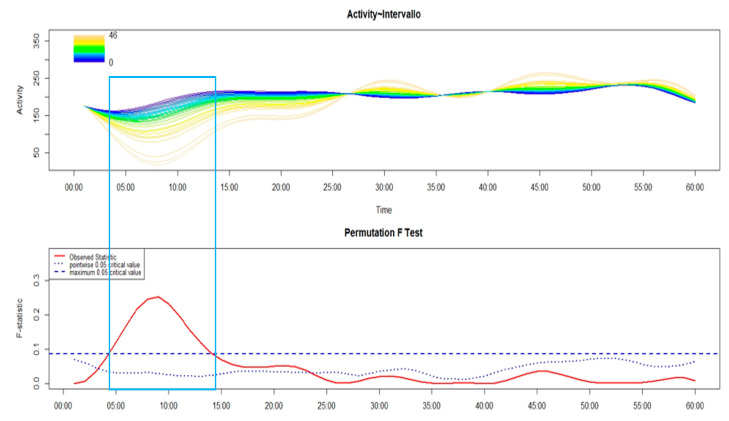
The upper panel of the FLM plot shows the functional forms of motor activity, minute-by-minute, over the first 60 min after sleep end plotted against the time interval, in minutes, between sleep end and the time at which patients with MIX-I remembered to perform the activity-based prospective memory task, with the dark colors pointing to a lower time interval while light colors show a longer time interval as reported in the plot’s color spectrum legend. The lower panel shows the results of the non-parametric permutation F test. The rectangle highlights the time window with significant differences in motor activity according to the time interval between sleep end and the activity-based prospective memory performance.

As shown in the following figures (from Figure 5, Figure 6, Figure 7, Figure 8 and Figure 9), the covariation in motor activity was examined in the first 60 min after sleep end against the time interval in minutes between sleep end and the time at which participants performed the activity-based prospective memory task; the darker colors point to a shorter time interval while light colors indicate a longer time interval.

As reported in Figure 5, it was observed in HC that those who remembered to push the event-marker button of the actigraph early (the dark colors), had higher levels of motor activity in around the first 16 min after sleep end compared to those who remembered to perform the activity-based prospective memory task late (the light colors).

Regarding the patients with SO-I (Figure 6), a higher level of motor activity was observed around 3 min after sleep end in those who remembered to push the event-marker button early after sleep end compared to those who performed the activity-based prospective memory task later.

With reference to patients with NM-I (Figure 7), those who performed the prospective memory task early after sleep end were characterized by a significantly higher level of motor activity between around 4 and 12 min after sleep end, compared to those who remembered to perform the prospective memory task later.

With a specific focus on MA-I (Figure 8), within the time interval between around 6 and 34 min after sleep end, patients who remembered to perform the activity-based prospective memory task early after sleep end were those with a significantly higher level of motor activity compared to those who performed the prospective memory task late.

Finally, when examining MIX-I only (Figure 9), a significant covariation in motor activity was observed according to the time taken to perform the prospective memory task within the time interval between around 3 and 14 min. In addition, a significantly higher level of motor activity was apparent in those who remembered to perform the prospective memory task early after sleep end.

## 4. Discussion

The main aim of this study was to examine for the first time the activity-based prospective memory performance upon the awakening (cognitive sleep inertia) and its association with motor sleep inertia in different phenotypes of insomnia disorder. Compared to the previous literature [10] that only focused on the efficiency of activity-based prospective memory performance, in different phenotypes of insomnia disorder, considering whether the task was performed or not, the current study adds an assessment of cognitive sleep inertia, i.e., the time interval between sleep end and the time participants actually remembered to push the event-marker button of the actigraph. Moreover, the present study also examined the relationship between cognitive and motor sleep inertia, considering the mean motor activity, minute-by-minute, in the first 60 min after sleep end in the morning.

Regarding cognitive sleep inertia, it was observed that a longer time interval was taken to perform the activity-based prospective memory task in patients with MIX-I and MA-I compared to HC (Figure 2), pointing to a more marked cognitive sleep inertia in patients with these phenotypes of insomnia. This pattern of results, in line with those previously reported by Occhionero et al. [10] who examined whether the task was performed or not, shows how the insomnia disorder is able to modulate the inertia of the activity-based prospective memory performance in the morning, but to a different extent according to the phenotype of the disorder. A potential explanation of this result is that sleep continuity, which is markedly impaired in patients with problems in the maintenance of sleep, may play a primary modulating role in cognitive sleep inertia. The fragmentation of sleep, typical of maintenance insomnia disorder, may suggest a modification of the physiological-cyclical alternation between NREM and REM sleep during the night; this could indirectly provide evidence in favor of the suitability of the sequential hypothesis [21,22], which underlines the relevance of the cyclic alternation between NREM and REM sleep for the formation of memory, with different stages of sleep playing different roles in this process. Within the theoretical framework of an active role being played by sleep in memory consolidation [23,24], it is therefore possible to suggest (first hypothesis) that a minor consolidation of the intention, due to a condition of sleep fragmentation, may lead to a more marked cognitive sleep inertia in MIX-I and MA-I phenotypes only, i.e., those with a more pronounced fragmentation of sleep.

Regarding the association between cognitive and motor sleep inertia, it was observed, both in HC (Figure 5) and different phenotypes of insomnia (from Figure 6, Figure 7, Figure 8 and Figure 9), that those who remembered to perform the activity-based prospective memory task early after sleep end (low cognitive sleep inertia) were characterized by a higher level of motor activity (low motor sleep inertia) than those who remembered to perform the prospective memory task later after sleep end. From our point of view, it is even more important to underline that the phenotype of insomnia disorder also seems to modulate the association between cognitive and motor sleep inertia, with a significant effect that spreads over a differing period of time. In particular, in absolute terms, this period of time is longer in MA-I (28 min) and MIX-I (11 min), compared to NM-I (8 min) and SO-I (1 min). Moreover, it is interesting to highlight that, contrary to HC (Figure 5), these significant associations between cognitive and motor sleep inertia start a few minutes after sleep end in each insomnia phenotype (from Figure 6, Figure 7, Figure 8 and Figure 9). Overall, the results on the association between cognitive and motor sleep inertia seem to suggest (second hypothesis) that a more marked cognitive sleep inertia may be due to a more pronounced motor sleep inertia, potentially seen as a byproduct of a lower restorative value of sleep due to its fragmentation.

In order to try to identify the most convincing hypothesis of the two previously put forward, it could be useful to take a look at the different time courses of motor sleep inertia among the different phenotypes of insomnia, as reported in Figure 4. It seems that the insomnia phenotype with the least marked motor sleep inertia is MA-I; this result could be explained considering that, since the sleep of this insomnia phenotype is markedly impaired in terms of continuity, these patients are already fully active after sleep end. The fact that MA-I represents the insomnia phenotype with the least marked motor sleep inertia does not seem to be in line with the second hypothesis, according to which the more marked cognitive sleep inertia was due to a higher motor sleep inertia. Therefore, this may lead to the speculation that the first hypothesis, which underlines the role of the altered cyclic alternation of REM and NREM sleep stages in the impaired consolidation of the intention, due to a condition of sleep fragmentation, seems to be more convincing.

Finally, we are also fully aware of a few limitations of the present study. Among them, we may quote the unbalanced number of patients that, following actigraphic quantitative criteria [10,12], were grouped into the different phenotypes of insomnia; specifically, the sample size of SO-I was smaller compared to the other groups. Moreover, another limitation that should be kept in mind is the age difference between patients and controls. There are additional limitations in this study, including the absence of both self-reported and objectively measured light exposure, which could have influenced sleep quality, activity levels, and cognitive function. Furthermore, the lack of objectively assessed motor activity over 24 h may have prevented a thorough evaluation of how circadian rhythm amplitude impacts the study’s outcome measures. Lastly, we may also mention that the insomnia phenotype of early morning awakening was not taken into account; however, we should also keep in mind that, in the case of this specific insomnia phenotype, it would have been difficult to set sleep end correctly.

## 5. Conclusions

Overall, this study has shown how the phenotype of insomnia disorder seems to modulate cognitive sleep inertia, with a more marked inertia in MA-I and MIX-I. It also highlights its association with motor sleep inertia, with a significant relationship that spreads over a longer time interval in MA-I and MIX-I.

## Figures and Tables

**Table 1:** Means and standard deviations of actigraphic sleep parameters in patients with different phenotypes of insomnia and healthy controls. Significant differences between groups marked with asterisk.

	SO-I	MA-I	MIX-I	NM-I	HC	F_4,112_	*p*
TST	437.86 ± 54.70	395.53 ± 48.80	418.05 ± 62.15	441.35 ± 52.39	428.90 ± 41	2.89	<0.05 *
SE	91.46 ± 2.71	85.07 ± 7.25	83.96 ± 11.14	94.49 ± 1.66	94.72 ± 1.64	18.95	<0.001 *
SOL	23.21 ± 8.56	8.69 ± 3.17	30.08 ± 21.98	7.66 ± 2.67	8.26 ± 2.82	25.50	<0.001 *
WASO	18.58 ± 10.66	62.91 ± 37.22	59.06 ± 50	16.92 ± 4.78	12.97 ± 5.39	19.43	<0.001 *
NA > 5	2.16 ± 0.36	4.25 ± 1.96	3.82 ± 1.87	1.83 ± 0.59	1.50 ± 0.49	26.11	<0.001 *
MA	14.80 ± 4.09	20.94 ± 7.97	21.49 ± 12.65	10.54 ± 2.47	10.62 ± 2.09	16.02	<0.001 *

Note: SO-I = sleep onset insomnia; MA-I = maintenance insomnia; MIX-I = mixed insomnia; NM-I = negative misperception insomnia; HC = healthy controls; TST = total sleep time (minutes); SE = sleep efficiency (%); SOL = sleep onset latency (minutes); WASO = wake after sleep onset (minutes); NA > 5 (number of awakenings longer than 5 min); MA = mean motor activity during the night (activity counts).

## Data Availability

The data are not publicly available and cannot be shared due to ethical issues.
